# Editorial: Informal STEM learning at home and in community spaces

**DOI:** 10.3389/fpsyg.2024.1383075

**Published:** 2024-03-13

**Authors:** Bradley Morris, Brenna Hassinger-Das, Jennifer DeWitt, Rachael Todaro

**Affiliations:** ^1^Learning Sciences and Educational Psychology, Kent State University, Kent, OH, United States; ^2^Psychology Department, Pace University, New York, NY, United States; ^3^University College London, London, United Kingdom; ^4^Department of Psychology, Temple University, Philadelphia, PA, United States

**Keywords:** informal learning, informal STEM learning, STEM learning environments, everyday learning activities, family learning environment

This Research Topic investigates how children's authentic, everyday experiences provide opportunities for STEM learning and engagement. Providing children with equitable opportunities to engage in, learn, and flex their STEM skills is critical because STEM drives new innovations across disciplines, accelerates discoveries, and finds creative ways to solve big challenges now and in the future (Fenechel and Schweingruber, 2010; Archer et al., 2022). But, what do we mean by informal STEM learning? We define “learning” broadly to include traditional definitions that focus on knowledge change and conceptual understanding, but we also include other areas supporting learning and connected to a more expansive definition, such as interest, engagement, and identity (see Fenechel and Schweingruber, 2010, for a detailed discussion). But why informal learning, in particular? This area is important because children spend only around 5% of their lives learning in formal settings (Falk and Dierking, 2010). Of that time, children are estimated to spend ~142 h per year in math instruction (nearly half of what is spent on Language Arts instruction; Phelps et al., 2012) and only a small portion of formal instructional time is spent learning about science (Falk and Dierking, 2010). Thus, the possibilities and opportunities for learning STEM skills outside of these traditional settings are substantial.

We proposed this Research Topic because despite significant interest in informal learning, particularly in STEM, the research is often disseminated through disparate journals, conferences and other outlets, which do not always share contributors or audiences. This Research Topic is an attempt to aggregate research from multiple fields that share overlapping interests and allow scholars from different fields to share their research in one place. The Research Topic explores unique opportunities to increase participation in STEM activities by sparking children's interest in science, providing meaningful connections to their lives, engaging children and their caregivers, and promoting STEM identities, or the belief that one can participate in STEM, whether inside or outside of the classroom or STEM “pipeline”.

The articles in this Research Topic investigate three key issues: (a) investigating how and where informal STEM learning occurs, (2) innovations in the measurement of informal learning, and (3) interventions to increase engagement in and knowledge related to STEM.

## Investigating how and where informal STEM learning occurs

How do families and children engage in STEM learning in homes and public spaces? The center of the informal STEM learning ecosystem is the family home. The first group of articles investigate the variations in the kinds of informal STEM activities in which families engage in their homes. Parental beliefs about the importance of STEM is a strong predictor of child outcomes (e.g., STEM careers; Rozek et al., 2017).

Silver et al. investigated the relation between parental beliefs about math (using the Home Numeracy Questionnaire), parent and child gender, and their influence on the frequency of informal math activities with toddlers. Although there were no differences in engagement based on child gender, mothers engaged in more activities than fathers. However, there were no differences in parental engagement when parents had strong beliefs about the value of math. Marcus et al. and Sobel and Stricker investigated family STEM engagement at home. Marcus et al. observed families via Zoom while they participated in a tinkering activity. Half of the families were instructed to create a story about the activity before tinkering whereas the other half were asked to begin the task. Families given the story prompt produced more STEM talk and more detailed reminiscence when compared to the tinkering only group.

Sobel and Stricker investigated the role of parent-child interactions in their children's hand washing behaviors and their causal understanding of germs. Parents and children either watched a handwashing demonstration or jointly participated in a handwashing activity. Children were less likely to use soap when washing their hands when their parents used more directive talk (e.g., setting goals) during the handwashing task, suggesting that providing children with more autonomy during the learning experience increases later engagement. Bae et al. report the results of a 5-year longitudinal study investigating how home science inputs, such as casual talk and science-related materials, influence children's science literacy. Science-related materials in the home were predictive of later science literacy but surprisingly, parent causal talk had a short-term effect and was not predictive of later science literacy. Msall et al. investigated parent attitudes about informal math learning opportunities in the home. A survey of 344 adults with 3- or 4-year-olds measured parent beliefs about what they considered the most effective ways to teach children about math and which approaches they used in their own homes. The results demonstrated a disconnect in that many parents reported using direct instruction but rated incorporating math into daily lives as the most valuable.

Public spaces such as museums and libraries provide unique opportunities for STEM learning. Leech et al. recorded family conversations in a science center and compared the amount of science talk initiated by fathers and mothers. The findings showed that fathers produced more science questions to their children and produced more wh-questions, which promoted more science discourse. Franse et al. report on a project to engage the public in complex social problems, or wicked problems, in science museums. Participants discussed issues in personalized medicine in a focus group and their responses were coded to measure interest in the topic. The findings suggest that exhibits in museums that convey the importance of the issue and build on general interest in science might be most effective in engaging the public in difficult societal questions.

## Innovations in the measurement of informal STEM learning

Accurately measuring informal STEM learning presents challenges because of the variation in where and how families engage with STEM content. To better capture children's everyday spatial behaviors (and how those behaviors relate to other aspects of development), Yang et al. developed the Everyday Spatial Questionnaire for Children (EBSQC) for parents to rate children's spatial behaviors. Their research found that the EBSQC significantly correlated with children's sense of direction, adaptive living skills, and cognitive skills.

Douglas et al. and Miller et al. all focused on early math in the home environment. Douglas et al. conducted a series of studies to evaluate measures of parents' knowledge about their children's early math skills—particularly numeracy and patterning. These beliefs likely influence their knowledge about early math and their efforts to support their children's math skills. Miller et al. also sought to evaluate the home environment, particularly for toddlers, using surveys, time diaries, and observations of math talk. They argued that using such diverse methodologies is necessary to measure separate components of the home math environment, including math activities and math talk, which both predict math skills in different ways.

Kominsky et al. and Weisberg et al. also introduced novel methods for measuring children's informal STEM learning–both featuring technology. Kominsky et al. introduced a mobile-based research app called “Talk of the Town” that is designed to capture children's informal STEM learning. “Talk of the Town” will be open-access and facilitate the collection of data from more diverse samples, since data collection will not be constrained by location. Similarly, Weisberg et al. took their research into a non-traditional location—a children's museum—using GoPro cameras. Children wore the GoPro cameras during a 10-min period while they interacted with museum exhibits, family members, and museum staff. Findings suggested the value of interacting with exhibits with caregivers and that children's learning benefitted the most from static (vs.s interactive) exhibits.

## Interventions to increase engagement in and knowledge related to STEM

The final group of articles describe interventions to promote learning and engagement through informal STEM activities. Gaias et al. evaluated a library-based program, Fun with Math and Science (FMS), that supports and enhances family STEM interactions. The FMS program includes information about child development, modeling interactions, and allowing families to practice interactions during activities. The results showed that families in the program engaged in more behaviors related to math and science learning than families who did not participate in the program.

Zucker et al. investigated the efficacy of home-based family STEM opportunities delivered virtually. Families were mailed materials to be used during virtual “funshops” in which families watched videos that provided engagement prompts (e.g., wh-questions) and instructions for using materials. The results provide an important caution about virtual delivery and suggest that virtual programming might be limited in its effectiveness because it requires substantial time and resources.

Haden et al. review research on the use of storytelling as a mechanism for enhancing STEM learning and engagement in Latine communities. The review provides evidence that the use of storytelling is a strengths-based approach that leverages funds of knowledge in Latine communities to link STEM learning with everyday activities. Íleri et al. review the evidence regarding how the affordances of toys influence the development of spatial reasoning. They identify toy features, most notably folding, that are most influential for proving interesting and challenging experiences for children. The authors provide recommendations for toy designers that will enhance spatial development through individual play and social interactions.

Makerspaces are popular in many public STEM spaces. Lukowski et al. investigated the impact of structuring makerspaces to include assembly-style making. Although some previous research has suggested that this might reduce creativity and engagement, the results from this study suggest that this approach helped novices feel more comfortable (and less overwhelmed), promoted tinkering, and supported family interactions.

One important facet of informal STEM learning is leveraging everyday experiences to make STEM learning meaningful and interesting. Wang and Walkington created a program in which students shared STEM questions with their peers derived from everyday environments and objects (e.g., Would a cylinder or bag hold more chips?). Students generated more complex questions and deeper explanations compared to more traditional assignments.

This Research Topic encourages reflection by depicting a broad variety of ways in which families engage in informal STEM as well as the wide-ranging contexts in which engagement occurs. It also provides a foundation for future investigations into informal STEM learning by outlining innovations in methodology and intervention. Novel instruments and creative approaches to measurement and data collection allow for better understanding of how informal STEM experiences influence STEM engagement for children and families. Finally, several of the articles provide examples of thoughtful interventions that increase informal STEM learning. By turning a lens on the 95% of children's time spent outside of formal learning contexts, the research within this issue makes a significant contribution toward increasing opportunities for effective STEM learning in everyday situations.

## Author contributions

BM: Writing – original draft, Writing – review & editing. BH-D: Writing – original draft, Writing – review & editing. JD: Writing – original draft, Writing – review & editing. RT: Writing – original draft, Writing – review & editing.
